# Florence Nightingale, O.M.: The Memorial Service at St. Paul's Cathedral

**Published:** 1916-02-19

**Authors:** 


### FLORENCE NIGHTINGALE, O.M. *1820-1910.*

### THE MEMORIAL SERVICE AT ST. PAUL'S CATHEDRAL.

The Queen, on the afternoon of Monday, the 14th instant, unveiled the Florence Nightingale Memorial in the Crypt of St. Paul's Cathedral. The Memorial is placed on the wall to the left, in the passage leading from Nelson's tomb to that of Wellington, and through the passage on the right are the graves of Wolseley and Roberts, with the tablets referring to them. Mr. Arthur G. Walker, the sculptor, who is also the sculptor of the Florence Nightingale statue in Waterloo Place, is to be congratulated on his most appealing and beautiful work. The Memorial is a low-relief in white marble, representing Florence Nightingale in half-length, wearing the familiar cap, bending over a wounded soldier, to whose lips she is holding a cup. The marble is surrounded by a rich alabaster frame, the inscriptions upon which are, above the plaque, "Blessed are the merciful," and below it, "Florence Nightingale, O.M.; born May 12, 1820, died August 13, 1910."

The ceremony was of the simplest. On either side of the passage were members of the Nightingale Memorial Committee, including, in addition to those mentioned specially hereafter, Lord Oranmore and Browne, Lord Knutsford, Sir Henry Burdett, Dr. Ogier Ward, and Miss Amy Hughes. There were also present the High Commissioners for New Zealand and South Africa; Sir John M'Call, the Agent-General for Tasmania; Lady Perley, representing the High Commissioner for Canada; the Lady Mayoress; Miss Macdonald, R.R.C., principal matron of the Canadian Nursing Service; Miss J. C. Child, representing the Matron-in-Chief of the Nursing Association of South Africa; Mr. Walter Bonham-Carter, secretary to the Nightingale Fund; Surgeon-General Carleton Jones; and Major-General J. W. Carson, C.B. The Archbishop of Canterbury and the Lord Mayor, who came in State, stood under the archway facing the Memorial, where they were joined by Her Majesty the Queen, with whom was Princess Mary, under escort by the Dean and clergy. The Archbishop of Canterbury, with his clear voice, was an admirable spokesman.

### The Archbishop's Tribute.

May it please your Majesty,—It is my privilege as spokesman of those who are here and of very many who are not here to ask your Majesty to unveil a monument of beauty and importance in itself, with a significance enhanced tenfold by the circumstances which, in the nation's life, surround this hour of its unveiling. More than half a century has passed since the dark and anxious winter of 1854, when, under the clear eye and the firm hand of a lady whose vision and capacity were on a par with her splendid devotion, chaos and mismanagement began to disappear from our Army hospitals, a new era of nursing was inaugurated, and the name of Florence Nightingale was on every lip. In a few short weeks the puzzled curiosity and the half-adulatory, half-critical surprise with which her enterprise was greeted had been merged in the universal acclaim of gratitude and praise, and into the modern life of "this troublesome world" a new benediction had been born.

For half a century we have thanked God for what Florence Nightingale has wrought and taught, but we did not know its range or its greatness until now. So it is fitting that your Majesty, on behalf of English womanhood, should unveil this monument in a year when, in the nation's need, tens of thousands of women are, with persistency of quiet devotion and a ministry of steadily increasing skill, following the path wherein "the lady with the lamp" was pioneer.

It is easy, or, rather, it is not easy, to measure what the difference might have been had that pioneer been a woman of unbalanced enthusiasm, however eager, or of mere plodding devotion, however praiseworthy. The lead she gave might easily have been discredited and therefore fruitless of result. But when to a buoyant faith, a courageous hope, and a large love were superadded the gifts of penetrating judgment, of potent personal influence, and of almost unrivalled administrative skill, the fruitfulness of the leadership was immediately secure. England and, through England, the whole world know now, to a degree they never knew before, in camp and hospital, on land and sea, the priceless value of the gentle deftness and the tender skill of trained womanhood both in peace time and when the horrors of war are at their worst.

We do well to set here in our Cathedral among our warriors' tombs the monument of one to whom we owe so much, and I ask your Majesty to make visible to all of us a beautiful and enduring reminder of the lessons of her life.

At the conclusion of the address Her Majesty, having unveiled the plaque, said, "I have great pleasure in unveiling this Memorial." The Queen then studied the memorial, and seemed to be won by the spirit of tenderness which breathed over the sculptor's beautiful creation. Her Majesty, having shaken hands with the Archbishop and the Lord Mayor, Lord Pembroke, the chairman of the Memorial Committee, presented Mr. Walker, the sculptor, to the Queen, who then shook hands with the members of the Committee on her left, and, asking where the tomb of Lord Roberts was, again returned through the archway past the Memorial to the right where it was placed with that of Lord Wolseley. Her Majesty was then escorted into the Cathedral and took her place under the dome during the special service, particulars of which will be found on page 464.

The space in the Crypt was very restricted, but there were present some who had shared Miss Nightingale's labours and been associated with them. Mr. Henry Bonham-Carter, who for fifty-three years was the close friend and adviser of Miss Nightingale, and the trusted secretary of the Nightingale Fund and Training School for Nurses.

Mr. Bonham-Carter, the brain of her organisations, was associated with Florence Nightingale and successive matrons of St. Thomas's Hospital from the commencement and throughout the growth of the movement for training nurses and improving their status. Although eighty-eight years of age he is still vigorous, and was keenly interested in the proceedings and also in his nurses who attended the service in the Cathedral. Mr. J. G. Wainwright, the treasurer of St. Thomas's Hospital, who for many years was associated with Florence Nightingale, and whose recovery after an operation has been welcomed by all hospital workers, in proportion to the closeness of their knowledge of the excellent work which he has done to render St. Thomas's Hospital efficient and adequate. Next to Mr. Bonham-Carter stood the president of the Royal College of Physicians, Dr. Frederick Taylor, whose whole working life has been associated with the medical school of Guy's Hospital, its re-creation and all the many events which together have placed Guy's Hospital, to-day, upon sure foundations, and made it the great school of medicine and of nursing which it undoubtedly is. Sir Wolfe Barry, the chairman of the Westminster Hospital, another famous school of nursing, who recently published a most masterly monograph upon its past and future and its removal from Westminster Broadway to a new site facing Clapham Common; Lord Pembroke, who succeeded his father, the first chairman of the Memorial Committee, as the representative of the late Sidney Herbert, who selected Florence Nightingale, sent her to the Crimea, and proved her staunch friend and supporter, as history records. Lord Pembroke had come from the trenches to St. Paul's in order to be present at the unveiling. He inherits, through his father's deep interest in Florence Nightingale and her work, the privilege of being trustee for the funds raised by the Memorial Committee, which have been devoted to the provision of pensions for nurses through the Trained Nurses' Annuity Fund. Lord Crewe, the Lord President of the Council, has shown the deepest interest in this Memorial from its earliest days. He has rendered valuable service as a regular attendant at the committees, and by his wise counsel and invaluable co-operation. He was accompanied by the Dowager Lady Pembroke, and by his wife, whose presence was welcome as an indication that she shares her mother the late Lady Rosebery's interest in everything which has to do with the uplifting, comfort, and help of the poorest and most defenceless. Miss Sidney Browne, R.R.C., the Matron-in-Chief of the Territorial Force Nursing Service, was the only woman representative of the Army present. Much comment and surprise were expressed at the absence of Miss Becher, Matron-in-Chief of Queen Alexandra's Imperial Military Nursing Service, who should have attended as the representative of the War Office. In her absence, explained by routine duty, inspecting, the War Office was unrepresented. There were also present Miss Lloyd-Still, the very able matron of St. Thomas's Hospital, who has been a diligent attendant at committees, and has done good service since the war in many directions; and Mr. G. Q. Roberts, the honorary secretary of the Memorial Committee, who has worked hard and laboured untiringly with gratifying results to make the Memorial a success.

We were glad to notice the presence in the Cathedral of the following matrons of London hospitals: Miss McIntosh (St. Bartholomew's), Miss Haughton (Guy's), Miss Montgomery (Middlesex), Miss Darbyshire (St. Mary's), Miss Bird (Great Northern Central), Miss Nevile (West London), Miss Rede (Hospital for Consumption, Brompton), Miss Row (East London for Children), and Miss Squier (Hospital for Women, Soho). The representatives from the provincial hospitals included the following matrons: Miss Sparshott, R.R.C. (Royal Infirmary, Manchester), Miss Musson, R.R.C. (Birmingham General Hospital), Miss Scott (Royal Sussex County Hospital, Brighton), Miss Herbert (Worcester General Infirmary), and Miss Bryan (late matron Northampton General Hospital). Miss Rosalind Paget (London Hospital and Central Midwives Board), Miss Cubitt and Miss Robinson (Incorporated Society of Trained Masseuses) also attended. One row of seats was entirely occupied by present Nightingale nurses, whilst some hundreds of sisters and nurses representing every branch of the nursing profession were also present. It was a remarkable and gratifying testimony to the national interest taken in Florence Nightingale and her work that the vast Cathedral was filled, without any special effort having been made to attract a congregation. Amongst the most generous contributors to the Memorial Fund were the officers and crews of His Majesty's ships and the officers and men of His Majesty's regiments. An Admiralty party of upwards of 100 sailors with an officer, and many wounded soldiers, represented the Services.

The service in the Cathedral was simple, and the anthem, beautifully rendered, went straight to the hearts of all present. We have attended many memorial services, but we do not remember one which could compare with this for its power to awaken the spiritual side of each member of the congregation, and to bring home directly to their hearts the force of the personal service rendered by the departed. It seemed to fill them with the spirit of Florence Nightingale and her teachings, which have done so much and will continue to do so much for the uplifting of the nursing profession and of all workers amongst the sick.

This account of a living memorial to the only woman whose life-work has been recognised by the distinction of the Order of Merit would be incomplete if it did not record the graciousness of Her Majesty the Queen, who entered fully into the spirit of the occasion and won all hearts. It was a great pleasure to recognise the lifting from Her Majesty's shoulders of the grave anxiety she has gone through since the King's accident, and to realise from the atmosphere which she shed around Her on this occasion the admirable way in which Queen Mary fulfils the duties of her high position.

